# RNA-Binding Proteins in *Trichomonas vaginalis*: Atypical Multifunctional Proteins Involved in a Posttranscriptional Iron Regulatory Mechanism

**DOI:** 10.3390/biom5043354

**Published:** 2015-11-26

**Authors:** Elisa E. Figueroa-Angulo, Jaeson S. Calla-Choque, Maria Inocente Mancilla-Olea, Rossana Arroyo

**Affiliations:** 1 Departamento de Infectómica y Patogénesis Molecular, Centro de Investigación y de Estudios Avanzados del IPN (CINVESTAV-IPN), Av. IPN # 2508, Col. San Pedro Zacatenco, CP 07360 México, D.F., Mexico; 2 Laboratorio de Inmunopatología en Neurocisticercosis, Facultad de Ciencias y Filosofía, Universidad Peruana Cayetano Heredia, Av. Honorio Delgado 430, Urb. Ingeniería, S.M.P., Lima 15102, Peru

**Keywords:** *Trichomonas vaginalis*, RNA-binding protein, posttranscriptional regulation by iron, iron, IRE-IRP system, HSP70, α-Actinin, Actin

## Abstract

Iron homeostasis is highly regulated in vertebrates through a regulatory system mediated by RNA-protein interactions between the iron regulatory proteins (IRPs) that interact with an iron responsive element (IRE) located in certain mRNAs, dubbed the IRE-IRP regulatory system. *Trichomonas vaginalis*, the causal agent of trichomoniasis, presents high iron dependency to regulate its growth, metabolism, and virulence properties. Although *T. vaginalis* lacks IRPs or proteins with aconitase activity, possesses gene expression mechanisms of iron regulation at the transcriptional and posttranscriptional levels. However, only one gene with iron regulation at the transcriptional level has been described. Recently, our research group described an iron posttranscriptional regulatory mechanism in the *T. vaginalis tvcp4* and *tvcp12* cysteine proteinase mRNAs. The *tvcp4* and *tvcp12* mRNAs have a stem-loop structure in the 5'-coding region or in the 3'-UTR, respectively that interacts with *T. vaginalis* multifunctional proteins HSP70, α-Actinin, and Actin under iron starvation condition, causing translation inhibition or mRNA stabilization similar to the previously characterized IRE-IRP system in eukaryotes. Herein, we summarize recent progress and shed some light on atypical RNA-binding proteins that may participate in the iron posttranscriptional regulation in *T. vaginalis.*

## 1. Introduction

Iron is an essential element for all living organisms and is involved in a broad range of relevant biological reactions. Iron is necessary for the assembly of Fe-S clusters in proteins and is an important component of heme-binding proteins including oxygen transport proteins, non-heme iron proteins, and ribonucleotide reductases [1,2]. Proteins that use iron as a cofactor are located in mitochondria, the cytosol, and the nucleus, and have different functions including electron transfer, ribosome maturation, DNA replication and repair, and cell cycle control [3,4,5,6,7,8,9,10,11,12].

**Figure 1:**
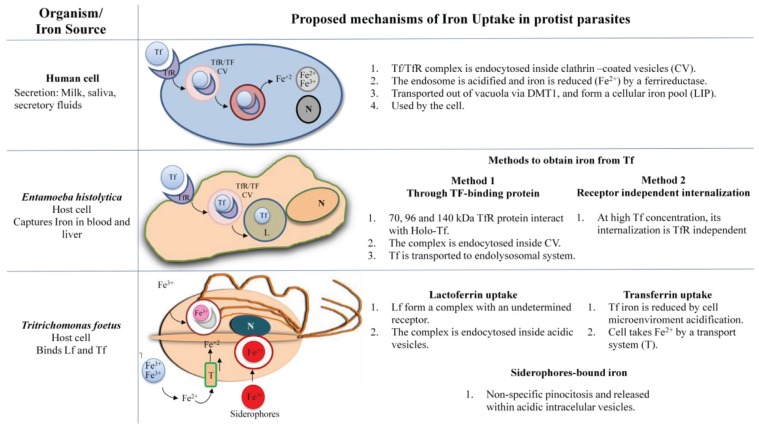
Possible mechanisms of iron uptake by human*, Entamoeba histolytica* and *Tritrichomonas fetus* cells from different iron sources. The mechanisms used to internalize iron are explained. Figures modified from Sutak R *et al.*, 2008 and Reyes-López M *et al.*, 2012 [18,19].

Iron is also an essential nutrient for multiple biological processes in protist parasites including survival, metabolism and virulence; many parasites require high iron concentration for living. However as part of the host evasion mechanisms against parasite invasion the extracellular free iron is withhold by the host cell and parasites have evolved to scavenge iron from multiple protein sources from the host. As the iron is predominantly intracellular and extracellular amounts of iron are very limited, and primarily it is attached to storage and transport proteins such as ferritin, transferrin (Tf), and lactoferrin (Lf) and iron-containing proteins such as hemoglobin (Hb); parasites have developed strategies to supply their needs. These mechanisms have been thoroughly reviewed in the e-book “The struggle for iron: pathogen *vs.* host” 2013 [13] and in the special number “iron and parasites”, 2015 [14]. For example, to acquire iron from Holo-Lf, parasites adopted several mechanisms such as: (1) The expression of Lf binding receptors or proteins with the capacity to bind directly the Holo-Lf (*Trichomonas vaginalis*) [15]; (2) Enzymatic degradation, by secreted proteases as in *Tritrichomonas foetus* and *Entamoeba histolytica*, to release iron from Holo-Lf; (3) Reducing enzymes, like reductases that promote the reduction of iron from the ferric to ferrous state (*Leishmania spp.*) and (4) Xenosiderophores that remove iron from Holo-Lf, which is captured by specific receptors [16]. The Tf protein found in serum and lymph binds Fe^3+^ with high affinity, and keeps the iron completely kidnapped. Eukaryotic cells, including human and parasitic protists have different mechanism for iron uptake (Figure 1).

*T. vaginalis* has also high requirements of exogenous iron (250–300 µM)*.* Iron is essential for its survival, metabolism, and multiplication in culture and regulates some of its virulence properties by known and unknown mechanisms. *T. vaginalis* uses multiple sources of iron from the host cells such as lactoferrin (Lf), hemoglobin (Hb) and heme. It has multiples iron uptake systems mediated by specific receptors: receptor for binding the cytochrome C, a 136 kDa receptor for binding the host holo-Lf and even uses the adhesins AP65 and AP51 as heme- and hemoglobin-binding proteins [17]. This parasite also internalizes ferritin, but not transferrin. Other important sources of iron are cells *i.e.*, erythrocytes and epithelial cells. Two erythrocyte-binding proteins of 12.5 and 27.5 kDa help *T. vaginalis* to acquire iron from Hb [17].

However, iron can be toxic at elevated cellular levels. Thus, regulatory systems have evolved to maintain non-toxic concentrations of cellular iron. Metazoan iron regulatory proteins have been extensively studied. However, few studies have investigated iron homeostasis in protozoan parasites, particularly those having a high dependence on iron to grow and express virulence factors. The review focused on recent progress made in understanding iron regulation in *Trichomonas vaginalis* that presents a high dependence on iron to grow and express virulence factors and a posttranscriptional iron regulatory mechanism that includes atypical hairpin structures in iron-regulated mRNAs and atypical RNA-binding proteins.

## 2. Iron Homeostasis: Intracelullar Regulation Mediated by the IRE/IRP System

Iron exists in two different redox states: the reduced ferrous form Fe(II) and oxidized ferric form Fe(III). At physiological oxygen levels, the Fe(III) is present in most of the biological complexes and reduction reactions are performed because only the reduced iron form can be used as a substrate for membrane transport, loading and release of iron from molecules such as ferritin, and for the synthesis of heme groups in diverse proteins [20,21]. Although iron is essential for all living organisms, its chemical properties as a transition metal demonstrates its toxic potential, generating oxidative stress due to its capacity to produce reactive oxygen species (ROS) through Haber-Weiss-Fenton’s reaction that produces damage in proteins, lipids, and nucleic acids [20].

Iron metabolism is finely regulated in a systemic manner and/or at the cellular level in higher eukaryotes. Iron homeostasis in mammals is regulated by three regulatory systems: (1) regulation of serum iron levels through Hepcidin-ferroportin proteins in the liver; (2) transcriptional regulation by Hypoxia inducible factor-2α (HIF-2α); and (3) intracellular iron homeostasis regulation by the IRE/IRP posttranscriptional machinery [22].

At the cellular level, both iron deficiency and iron overload can cause cellular damage. Thus, iron uptake, storage, and cellular distribution must be controlled to prevent an excess of iron that causes ROS production or a deficiency of this element that does not allow the metabolic demands of the cell to be met [23,24,25,26,27,28]. The cellular posttranscriptional iron regulation is mediated by cytoplasmic iron regulatory proteins (IRPs) that recognize iron-responsive elements (IREs) located in specific mRNAs. This mechanism was previously described for *ferritin* (*fer*) and *transferrin receptor* (*tfR1*) mRNAs. At low iron concentrations, IRPs are active and bind the five IREs located at the 3'-UTR of the *tfR1* mRNA, inhibit its degradation, and generate an increase in the amount of the TfR1 protein and, consequently, in the uptake of iron. Binding of IRPs to the IRE located at the 5'-UTR of the *fer* mRNA inhibits its translation and causes a decrease in iron storage in cells [4,29,30]. At high iron concentrations, IRP-1 inserts a 4Fe-4S cluster, acts as an aconitase enzyme, and loses its RNA-binding activity [23], whereas IRP-2 is targeted for proteosomal degradation through an E3 ubiquitin ligase complex [31,32]. An interaction between FBXL5 (one of the proteins of the E3 ubiquitin ligase complex) and IRP-2 or IRP-1 is iron dependent, and IRP-2 ubiquitination increases by the presence of FBXL5 [31,32]. Moreover, RNA interference strategies against FBXL5 demonstrate the relevance of the FBXL5 protein in the iron-dependent degradation of IRP-2 and apo-IRP-1. Interestingly, the FBXL5 protein has a hemerythrin-like domain that binds iron and that is degraded through the proteosome pathway when the cell grows under low iron conditions [32].

### 2.1. IRPs and the Aconitase Family: General Characteristics and Functions

IRPs are members of the aconitase family, a five-branched tree of Fe-S cluster-dependent hydrolases that catalyze the isomerization of β-hydroxy acid metabolites [33,34]. Active aconitases require a 4Fe-4S cluster, where three iron molecules attached to cysteine side chains participate in the isomerization reaction. Structural studies of mitochondrial aconitase revealed the presence of four domains, three of which are involved in the association to nestle the Fe-S cluster, while the fourth is tethered through a ~60 amino acid long linker [4,29]. IRPs are the proteins responsible for the iron protective system. Two IRPs have been identified in vertebrate cells: IRP-1 and IRP-2. The crystal structure of IRP-1 revealed that is a cytoplasmic (c-aconitase) enzyme with similarity to mammalian mitochondrial aconitase [35]. The Fe-S cluster- and substrate-binding residues are conserved and the active site is basically the same. IRP-1 and IRP-2 share ~60% identity at the amino acid level; however, IRP-2 does not display aconitase activity possibly due to the number of amino acid substitutions in the active site that may interfere with the Fe-S cluster assembly [35,36]. IRP-1 has a molecular mass of 98 kDa, whereas IRP-2 has a higher molecular mass of 105 kDa due to the 73 amino acid insertion in domain 1 of IRP-2 that could be responsible for the different pattern of affinities to IREs, iron-dependent oxidation, ubiquitination, and proteosomal degradation lost through evolution [4,36,37,38,39,40].

The relevance of the IRE-IRP machinery and its components was exposed by observing that animals lacking both alleles of *irp-1* and *irp-2* are not viable, supporting the essential role of IRPs in early development before embryo implantation [41]. In contrast, when mice lack only one of the IRP-coding genes, they remain viable, suggesting that the expression of one IRP can compensate for the lack of the other and showing the redundancy of functions between them [42]. Conditional deletion of both *irp* genes in hepatocytes compromised Fe-S cluster formation and heme synthesis and impaired mitochondrial functions, affecting its capacity to maintain the respiratory function [43]. Although both IRPs are ubiquitously expressed, recent studies in animal models have revealed that IRP-1 particularly contributes to systemic iron homeostasis and erythropoiesis regulation and has an important function in the pulmonary and cardiovascular systems [44,45]. Meanwhile, IRP-2 functions are primarily in the central nervous system and its deficiency is associated with neurodegenerative symptoms and microcytic hypochromic anemia [46,47,48]. Recent reviews have addressed in depth the physiological implications of the IRP/IRE regulatory system in mammals [21,49].

### 2.2. IREs, mRNA Stem-Loop Structures: Sequence and Location

**Figure 2:**
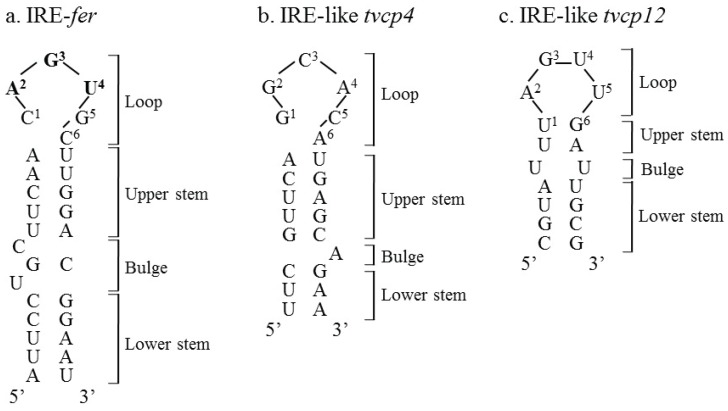
Comparison of the secondary structures of (**a**) the consensus iron-responsive element (IRE)-*fer* located at the 5'-UTR of the human ferritin heavy chain mRNA with (**b**) the *Trichomonas vaginalis* IRE-like *tvcp4* located at the 5' coding region of the cysteine proteinase 4 (*tvcp4*) mRNA and (**c**) IRE-like *tvcp12* located at the 3'-UTR of the cysteine proteinase 12 (*tvcp12*) mRNA. The important regions previously identified in the IRE structures are shown. Bold letters correspond to loop apex nucleotides. The figure was modified from Torres-Romero and Arroyo (2009) [61].

IREs are *cis*-acting mRNA stem-loop structures that are present in the 5'- or 3'-UTR of target mRNAs and that interact with IRPs to regulate the expression of several proteins involved in iron metabolism, heme synthesis, or the tricarboxylic acid cycle [21,36,50,51]. IREs are ~30-nucleotide (nt) structures folded into two RNA helices that are separated by a mid-helix cytosine residue bulge and a six nucleotide apical loop structure with the consensus sequence. Two optimal loop sequences have been identified, *i.e.*, the consensus sequence 5'-CAGUGN-3' (C1G5), where the N is usually a U, C, or A (never a G) residue and where the first C and the fifth G are believed to form a base pair that stabilizes the structure [24,50,52,53]; and the sequence 5'-UAGUAN-3' (U1A5), which is less common [54]. The IRE stem is divided into an upper helix between the C8 bulge and the terminal loop and a lower helix below the hinge (Figure 2). A phylogenetic analysis of approximately 150 eukaryotic IRE-containing mRNA sequences revealed high sequence conservation among them and suggested that IRE-*fer* represents the ancestral version of this type of translational control and the IRE structure was adopted by other genes during the evolution of higher animals [55].

The most studied IREs are located at the 5'-UTR of the *ferritin* (Figure 2a), *ferroportin*, *c-alas* and *hif-2α* mRNAs and at the 3'-UTR of the *transferring receptor* (*trf*) and *divalent metal transporter 1* (*dmt1*) mRNAs. The IRE-IRP regulatory network was systematically defined based on a transcriptome-wide scale of the IRP-1-IRE and IRP-2-IRE messenger ribonucleoprotein complexes from five murine tissues. The complexes were immunoselected, and the mRNA composition was determined using microarrays, which identified 35 mRNAs with specificity for IRP-1 or IRP-2 [56]. The identified mRNAs are divided into three different groups: the first class can recognize both IRPs and the other two classes are IRP specific. The mRNAs encode proteins involved in different cellular function that include metal ion-binding proteins, transferases, ligases, helicases, and transcription or DNA-binding factors. Considering the nature of the proteins identified, particularly the “metal ion-binding” category, Sanchez *et al.* (2011) [56] suggested extensively fine tuned coordination of iron/zinc metabolism pathways. The mRNAs that immunoprecipitate with IRP-2 contain IREs with the motif 4CN(5)CCGUG(A/U/C). Previously, *in vitro* assays demonstrate that IREs with the loop sequence UAGUAC have specific binding for IRP-1, that IREs with the loop sequence CCGAGC have specific binding for IRP-2 and that IREs with the sequence GAGUCG have specific binding for both proteins. The most conserved IRE sequence is CAGUGC [57] (Figure 2a). Although the majority of IREs are found in UTRs, IREs are also found in coding regions such as in *glycogenin*, a scaffold protein for glycogen synthesis; *thymidylate synthases*; and human *dihydrofolate reductase* mRNAs [56,58,59] as well as the atypical IRE-like hairpin structure found in the coding region of the cysteine proteinase 4 (*tvcp4*) mRNA of *Trichomonas vaginalis* [60] (Figure 2b).

### 2.3. IRE-IRP Interaction: A Key Step for the Posttranscriptional Regulation Mediated by Iron

Mutations within the first five nt of the loop are known to reduce IRP binding [62]. IRPs lack a common RNA-binding motif but still recognize a broad range of IRE stem-loop structures with picomolar affinity. The crystal structures of complexes formed between IRE-*trf* and IRE-*fer* with the IRP-1 protein revealed several important characteristics about the specificity of the RNA-protein interaction. The characteristic secondary structure adopted by IRP-bound IREs showed two separate regions for recognition. The first region is a terminal loop with the conserved sequence CAGUGN in the IRE structure and with a central AGU triplet in the apex. The second region corresponds to the mid-stem bulge with the conserved C8 (Figure 2a). The C8 hinge region forms multiple interactions with IRP-1 [63,64]. IRP-1 interacts with IRE through two specific sites: the terminal loop-binding cavity and the pocket for bulged C8. Each site participates in all types of RNA-protein interactions, particularly hydrogen bonds that are responsible for binding specificity. The crystal structural studies identified many of the contacts between IRP-1 and IRE. A rational construction of IRP-1 mutants focused on five residues (Arg269, Ser371, Lys379, Ser681, and Asn685) that allowed the contribution of each residue to complex stability to be determined. Three residues are located in the terminal loop-binding cavity and the other two residues are located in the C8 binding pocket. Although all the mutants have aconitase activity, the mutants in Arg269, Ser371, Lys379, and Ser681 residues showed significantly impaired interactions of IRP-1 with IRE-*fer*, causing a reduction in binding affinity that ranged from 8-fold to more than 100-fold and confirming the relevance of these residues in RNA-protein interactions [65]. In the IRE-IRP complex, the terminal loop-binding cavity showed the following interactions: Arg269 with U17, Ser371 with A15, and Lys379 with G16 (the central AUG triplet of the apex). The C8 bulge region involves only one unpaired nucleotide; however, any substitution at the position 8 would be unfavorable. Selezneva *et al.* (2013) [65] determined that the residues involved in the specific interaction of IRP-1 with RNA are not required for aconitase activity. Although the terminal loop and the C8 bulge regions of IREs are considered the most important for interactions with IRP-1, Goforth *et al.* (2010) [66] demonstrated that the upper stem-loop region proximal to the terminal loop plays a minor role in helping to stabilize the complex.

As we mentioned previously, IREs located at the 5'-UTR regulate mRNA translation under low iron conditions by controlling ribosomal binding into mRNAs and protein accumulation. The first step in protein synthesis is the eIF4E recognition and binding to the 5'-mRNA cap. eIF4E is one of the translation elongation factors that includes the small subunit of eIF4F and is responsible for recruiting the elongation factor eIF4A, an RNA-dependent ATPase that unwinds the secondary structure within the 5' non-coding region to allow the 40S ribosomal scanning. Metal-binding (Fe) could destabilize the IRE-RNA/IRP-1 complex and enhance the stability of the IRE-RNA/eIF4F complex. To understand the relative importance of the kinetics and stability of the RNA-protein complexes, the rate of RNA-protein complex assembly and dissociation for IRE-RNA with IRP-1 or eIF4F proteins was measured. Khan *et al.* (2014) [67] determined that the IRE-RNA/IRP-1 complex has a shorter half-life than the IRE-RNA/eIF4F complex, suggesting that both the rate of assembly and the stability of the RNA-protein complexes play key roles in immediate cellular responses to iron concentrations.

### 2.4. Atypical IREs and IRPs

Numerous studies have reported several atypical IREs that interact with typical IRPs that do not have consensus sequences, but are capable of interacting with IRPs in a specific manner. Although the great majority of the atypical IRE sequences are mutants compared with the canonical sequence, some IREs occur naturally as is the case for the stem-loop structure identified in the mitochondrial 75 kDa subunit of complex I reported by Lin *et al.* (2001) [68]. The amount of the 75 kDa protein is modulated by iron, whereas its mRNA level showed minor changes related to iron concentration. RNA-protein gel shifting assay (REMSA) demonstrated that the atypical IRE-75 recognized a cytoplasmic protein depending on the iron status and identified a putative IRP (75-BP) able to interact with IRE-*fer* [68]. Other atypical IREs have been identified in mRNA encoding iron metabolism related proteins or heme-containing proteins [61] and some IRP homologs have been reported in mammals, birds, invertebrates, and bacteria [4,25,69,70,71,72].

#### *IRE-IRP Iron Regulation in Protists* 

Iron is essential for growth of many protists such as *Entamoeba*, *Leishmania*, *Plasmodium*, *Naegleria*, *Toxoplasma*, *Trypanosoma*, *Tritrichomonas*, and *Trichomonas*. Although the IRE-IRP regulatory system has been extensively described in higher eukaryotes, only few IRE-like structures and IRP-like proteins have been identified and studied in the protists to date.

Using cytoplasmic extracts from *Leishmania tarentolae*, Meehan *et al.* (2000) [73] detected a protein that specifically interacts with a mammalian IRE-*fer*. REMSA indicated that the mobility of the mammalian IRE/IRP complex differs from that of the mammalian IRE/*L. tarentolae* protein complex, suggesting differences in the structure and conformation of the bound complex. Cloning and characterization of an aconitase closely related to animal IRPs and plant aconitases were also reported in another kinetoplast, *Trypanosoma brucei* [74]. Immunofluorescence analysis revealed that the kinetoplast aconitase showed dual subcellular localization, suggesting that it not only participates in the mitochondrial Krebs cycle but also may have a different function in the cytoplasm, probably acting as IRP.

*Plasmodium falciparum* resides within human erythrocytes (RBCs) and uses their content for its metabolic needs, particularly hemoglobin, an iron-containing protein. Inside RBCs, *P. falciparum* metabolizes hemoglobin and detoxifies heme together with iron into hemozoin. *P. falciparum* has aconitase activity that facilitates the conversion of citrate to isocitrate through the intermediate *cis*-aconitase, in the tricarboxylic acid (TCA) cycle. Loyevsky *et al.* (2001) [75] cloned and sequenced a cDNA encoding a putative IRP (PfIRPa) with 47% identity to human IRP-1 and 40% to IRP-2. PfIRPa is ~103 kDa, recognizes mammalian IREs, is localized in the plasmodial compartment of infected RBCs, and has aconitase activity [75,76]. The plasmodial counterparts of this mechanism, hairpin-loop IREs, were also identified and found capable of specifically binding to the recombinant and endogenous malarial PfIRPa protein. Three IREs were found in the mRNA of a large hypothetical protein with unknown function encoded by a gene located in chromosome 13, in the mRNA of a small hypothetical protein; and in the coding sequences of the plasmodial genes *pfb0320* and *pfb0325c* [77]. Although the functions of their gene products remain unknown, they were identified as biocrystallized products of the heme moiety detoxification system [78].

*Entamoeba histolytica*, an enteric protozoan parasite capable of invading the intestinal mucosa and spreading to other organs uses iron as an important factor for growth, adherence, and virulence induction. The iron-dependent differential expression of mRNA encoding a 26 kDa hemoglobin-binding protein (*ehhmbp26*), a protein involved in iron acquisition, was also reported [79]. The existence of mRNAs with IRE-like hairpins in this organism is being experimentally analyzed. Searching the amoeba genome Hernández-Peña *et al.*, 2015 found sequences exhibiting stem-loop structures in some mRNAs. The ability of one IRE-like structure to form RNA-protein complexes was functionally analyzed by REMSA and crosslinking assays, and findings support the possible existence of an IRE-IRP-like regulatory system in *E. histolytica* (Hernández-Peña *et al.*, 2015, under revision).

## 3. *Trichomonas vaginalis* 

*Trichomonas vaginalis*, a flagellated protist parasite, is the causal agent of human trichomoniasis, the most common non-viral sexually transmitted infection worldwide. Trichomoniasis is a chronic infection that leads to vaginitis in women or to urethritis and prostatitis in men. Trichomoniasis predisposes individuals to HIV/AIDS and cervical and prostatic cancers and is responsible for pneumonia, bronchitis, and oral lesions in immunocompromised patients [80,81,82]. Trichomoniasis is also associated with preterm delivery, low birth weight, pneumonia, and mental retardation in newborn babies, increasing infant mortality [80,81,82,83].

**Figure 3:**
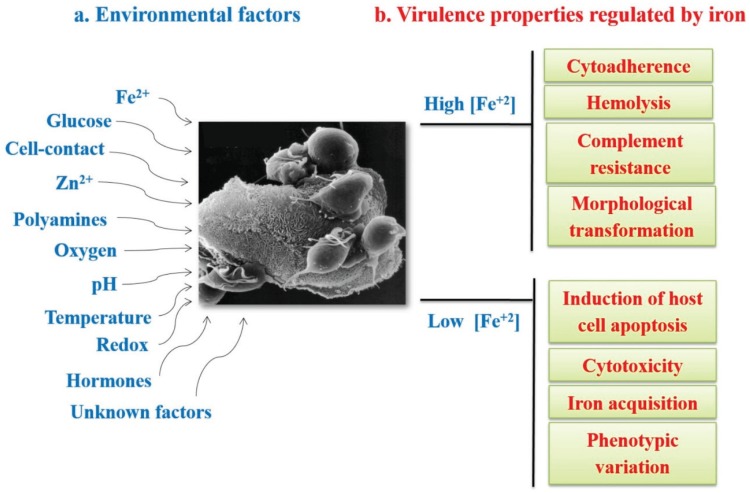
Environmental conditions affecting gene expression (**a**) and virulence properties differentially modulated by iron in *T. vaginalis* (**b**). (**a**) Environmental factors that influence gene expression and affect virulence attributes: iron, glucose, cell contact, zinc, polyamines, oxygen, pH, temperature, redox, hormones, and other unknown factors; (**b**) Virulence properties differentially-regulated by iron. ↑Fe^2+^: virulence properties enhanced under high iron conditions. ↓Fe^2+^: virulence properties enhanced under low iron conditions.

Trichomoniasis is a gender-related infection primarily affecting women, whereas the majority of men are asymptomatic, suggesting that a differential scenario may be related to differences in the urogenital microenvironments affecting trichomonad pathobiology. *T. vaginalis* responds to different environmental changes such as temperature, pH, oxygen, iron, glucose, polyamines, zinc, host immune responses, interaction with different host cells, and other unknown factors, modulating the expression of multiple genes, including those encoding virulence factors necessary to maintain a chronic infection ([17,80,84,85,86,87,88,89], Miranda-Ozuna *et al.*, 2015, under revision) (Figure 3a).

*T. vaginalis* has one of the largest genomes among protists, at ~160 Mb and with ~60,000 predicted coding genes [85], ~30,000 of which are expressed under different environmental conditions [87,88,89]. Analysis of ~100,000 expressed sequence tags (ESTs) generated by a collaborative work between several laboratories, revealed that *T. vaginalis* can differentially express its genes, depending on the environmental conditions. The EST sequences were obtained from parasites cultured under fourteen defined conditions related to the cell cycle, growth, starvation, and pathogenesis. The EST data are available in the TrichDB genome sequence database (http://www.trichdb.org) and in the Chang Gung University TvXpress database (http://www.TvXpress.cgu.edu.tw). *T. vaginalis* has a highly repetitive genome (at least 65%) with several multigene families such as the cysteine proteinase (CP) family, with more than 220 peptidase-coding genes that generate one of the most complex degradomes among protist parasites. An *in silico* analysis of genes implicated in trichomonal pathogenesis, at transcription and proteomic levels revealed that not all of the CP-encoding genes are expressed [17].

### 3.1. Iron in the Biology of Trichomonas vaginalis

One of the most studied environmental factors that influence the expression of virulence factors in *T. vaginalis* is iron. *T. vaginalis* requires iron as an essential element for survival, growth, metabolism, and multiplication in culture with high requirements of exogenous iron at concentrations between 250 to 300 µM [90,91]. *T. vaginalis* infects the urogenital tract, surviving and establishing an infection in the vaginal environment where the iron concentration is constantly changing under the influence of the menstrual cycle. Thus, both the survival and establishment of an infection will depend on the ability of *T. vaginalis* to adapt to the microenvironmental changes [87,88,89,91,92]. Iron stimulates several virulence properties such as cytoadherence, hemolysis, and complement resistance, but reduces cytotoxicity and host cell apoptosis induction, playing key roles in the host-parasite interaction. Iron modulates the expression of several virulence factors in *T. vaginalis*, including those encoding adhesins that are up-regulated by iron and those encoding several cysteine proteinases (CPs) that are differentially-regulated by iron (Figure 3b) [17,91,92].

Although few CP-encoding genes are expressed under different environmental conditions, CPs play important roles in the virulence of trichomonads. CPs participate in cytoadherence [17,93,94,95,96], cytotoxicity [97,98,99], hemolysis [100,101], and complement resistance [102], as well as disruption of the host cell membrane cytoskeleton [103] and degradation of the secretory leukocyte protease inhibitor [104], extracellular matrix proteins, and human immunoglobulins [105,106]. Interestingly, the expression, proteolytic activity, and surface localization of certain trichomonad CPs are differentially modulated by iron and other environmental factors [17,61,86].

Morphological and proteomic studies focused on *T. vaginalis* grown in the presence or absence of iron showed changes in the shape and proteome profile depending on the iron concentration. Approximately 600–640 and 540–570 spots were detected in gels with protein samples from *T. vaginalis* grown under iron-rich and iron-depleted media, respectively. The differentially expressed proteins were identified by mass spectrometry (MS) [107]. Among the proteins expressed by parasites cultured in iron-depleted medium, 12 were up-regulated, 19 were down-regulated, 11 had their expression abolished, and three had their expression induced. Actins, a Rab-GTPase, two 70 kDa heat-shock proteins (HSP70), and several CPs were among those up-regulated and induced proteins in *T. vaginalis* collected from iron-depleted cultures. Expression of multiple actin genes in *T. vaginalis* seems to suggest a selective advantage towards the parasite’s adaptation to different environments. The down-regulated proteins included several CPs and a pyruvate ferredoxin oxidoreductase (PFO) [107].

Using protease-resistant parasite extracts and MS analysis, we identified 27 protein spots: in the *T. vaginalis* active degradome: 13 were identified as unique proteins, including nine CPs, seven cathepsin L-like proteins (TvCP1, TvCP2, TvCP3, TvCP4, TvCP4-like, TvCP12, and TvCPT that corresponds to TvCP39), and two asparaginyl endopeptidase-like or legumain-like CPs, (TvLEGU-1 and a legumain-like CP that corresponds to TvLEGU-2) [99,108,109].

### 3.2. Iron Gene Expression Regulation in T. vaginalis

During infection *T. vaginalis* needs to quickly adapt to environmental changes, by regulating its gene expression at the transcriptional, posttranscriptional, or posttranslational level, through the dynamics and stability of the highly regulated formation of DNA- or RNA-multiprotein complexes that will determine which genes should be expressed or silenced. Iron-induced gene expression regulation is poorly understood in *T. vaginalis*. As the mechanism of RNA-binding protein-mediated posttranscriptional regulation by iron is the subject of this review, we begin by reviewing the other types of iron regulation. Table 1 and Table 2 show summaries of virulence factors expressed under high and low iron concentrations, respectively and the putative regulatory mechanisms involved in their expression.

**Table 1:** Genes and proteins up-regulated under high iron conditions: Functions and types of regulation.

Gene or Protein ID Number or NCBI Accession Number	Function	Type of Regulation	References
**AP23 ^Uk^**	Adhesin/virulence factor involved in cytoadherence	ND	[92,110,111]
**AP33** TVAG_047890	Adhesin/ α-subunit of succinyl coenzyme synthase/virulence factor involved in cytoadherence	ND	[85,92,110,111,112]
**AP51** TVAG_259190	Adhesin/β-subunit of succinyl coenzyme synthase/virulence factor involved in cytoadherence	ND	[85,92,110,111,112]
**AP65-1** TVAG_340290	Adhesin Malic Enzyme/virulence factor involved in cytoadherence	Transcriptional level	[85,92,110,111,112,113,114,115,116,117,118]
**AP120** TVAG_198110	Adhesin/Pyruvate: Ferredoxin Oxidoreductase/virulence factor involved in cytoadherence	Transcriptional level Posttranslational level ^G^	[85,86,111,112]
**BspA-like** TVAG_397210 TVAG_441420	Adhesin/Cytoadherence Host immune evasion	Transcriptional level	[85,86,112]
**TvCP4** TVAG_467970	CP/Hemolysis	Posttranscriptional level ^IRE/IRP^	[60,61,101,112]
**TvCP62 ^Uk^**	CP/Cytoadherence	ND	[105]
**TvENO-1** TVAG_464170	FN receptor Enolase/Cytoadherence	Transcriptional level	[85,86,112]
**TVF ^Uk^**	Cytolytic effector 250 kDa/Cytolytic activity	ND	[86]
**TvGAPDH** TVAG_146910	Plasminogen receptor Glyceraldehyde-3-Phosphate Dehydrogenase/Cytoadherence	Transcriptional level	[80,84,86,112]
**TvLEGU-1** TVAG_426660	CP/Cytoadherence	Transcriptional level Posttranslational level ^PG^	[85,96,108,109,112]
**TvLIP** AY870437	Triacylglycerol Lipase/Hemolysis	ND	[85,86,112]
**TvLPG ^Gl^**	Lipophosphoglycan/Cytoadherence and Cytotoxicity	Posttranslational level ^G^	[85,86,119]
**Ubiquitin** TVAG_097660 TVAG_264700	Ubiquitination	ND	[85,107,112]
**EF1α** TVAG_067400 TVAG_463940	Elongation factor 1α/Translation	ND	[85,107,112]
**Myb-3** TVAG_252420	MYB-like transcription factor	Transcriptional level Posttranscriptional ^P^ level	[112,113,116,117,118]
**TvACTN1** TVAG_156680	α-Actinin 1/Actin binding protein	Transcriptional level	[85,120]
**TvACTN2** TVAG_190450	α-Actinin 2/Actin binding protein/ RNA-binding protein	Transcriptional level	[85,120] This article
**TvACTN3** TVAG_239310	α-Actinin-3/Actin binding protein/RNA-binding protein	Transcriptional level	[85,120] This article
**TvHSP70** TVAG_044510 TVAG_139320 TVAG_092490	Chaperone/RNA-binding protein	Transcriptional Level Posttranslational level ^U^	[85,112] This article

^P^: phosphorylation; ^G^: glycosylation; ^IRE/IRP^: regulated by IRE/IRP-like mechanism; ^ND^: not determined; ^U^: possible regulation through the ubiquitination pathway; ^Uk^: unknown genes; ^Gl^: an exception, TvLPG is a surface glycolipid.

**Table 2:** Genes and proteins up-regulated under low iron conditions: Functions and type of regulation.

Gene or Protein ID Number or NCBI accesion Number	Function	Type of Regulation	References
**BspA-like** TVAG_093850 TVAG_299910	Adhesin/Cytoadherence Host immune evasion	Transcriptional level	[85,86,112]
**P270** AAD40228.1 TVAG_379560	Membrane Molecule/Phenotypic variation	Posttranslational ^P^	[80,121]
**TvCP12** TVAG_410260	CP/Cytotoxicity	Posttranscriptional level ^IRE/IRP^	([85,86], our unpublished data)
**TvCP30^M^**	CP/Cytoadherence Protein Degradation	ND	[61,85,93,94,95,105]
**TvCP39** TVAG_298080	CP/Cytotoxicity Igs Degradation	Posttranslational level ^GC^	[85,86,98,99,105]
**TvCP65** TVAG_096740	CP/Cytotoxicity	Transcriptional level Posttranslational level ^C^	[85,86,97,105]
**Myb-2** AY948337 TVAG_211210	MYB-like transcription factor	Transcriptional level	[85,113,115]
**TvHSP70** TVAG_163000	Chaperone	ND	[85,107]

^P^: phosphorylation; ^G^: glycosylation; ^IRE/IRP^: regulated by an IRE/IRP-like mechanism; ^ND^: not determined. ^U^: Possible regulation through the ubiquitination pathway; ^C^: regulation by the interaction with cystatin TC-2 [122]; ^M^: the 30 kDa band with proteolytic activity is formed by up to 10 spots with proteolytic activity that correspond to several gene products [61,86,93,95].

#### Transcriptional Regulation by Iron in *T. vaginalis*

Although the iron-induced mechanisms that regulate gene expression are poorly understood in *T. vaginalis*, EST analysis of the genomic database or transcriptomic assays suggests that gene expression is highly regulated by stringent differential transcription rate control [85,112,123,124]. Although, the knowledge of basal transcriptional regulation remains limited, primer extension assays have helped to identify the transcription start sites (TSSs) of several genes [124]. A study of the upstream sequences of protein coding genes revealed that ~75% contain a metazoan initiator-like element (Inr); a highly conserved sequence surrounding the TSS, TCA+1Py(T/A) that is structurally and functionally similar to its metazoan counterpart [123,124,125]. The *T. vaginalis* Inr sequence is responsible for TSS selection and is recognized by a 39 kDa protein (IBP39) that acts as a bridge between the Inr sequence and the RNA polymerase II pre-initiation complex to initiate basal transcription [126].

The Inr sequence is contained in the core promoter recently described as motif 1 by Smith *et al.* [123,125]. Four other motifs (motifs 2–4) were also identified in the 5'-upstream region of *T. vaginalis* genes that lack the typical Inr sequence (~25% of genes in the *T. vaginalis* genome). Only motifs 3 and 5 have been also studied thus far. Motif 3 resembles the metazoan Myb recognition element (MRE) and is recognized by the nuclear M3BP protein, a Myb-like protein with a similarity range of 40%–52% with Myb proteins. Additionally, motif 5 shows some degree of similarity with motif 1 [123,124,125,126].

Iron-inducible transcription has only been described for the *ap65-1* gene that encodes the AP65 adhesin, a 65 kDa surface protein with sequence homology to malic enzyme, which is involved in cytoadherence [86,92,110,111,113]. Briefly, the *ap65-1* has an iron-responsive promoter that contains a single Inr and other closely spaced regulatory elements including a T-rich sequence, and several MREs. MRE-1/MRE-2r, and MRE2f are recognized by three Myb transcription factors, TvMyb1, TvMyb2, and TvMyb3, which are responsible for *ap65-1* regulation [113,114,115,116,117,118]. In recent years, Tai’s research group demonstrated that TvMyb1 and TvMyb2 proteins show antagonistic effects on the basal and iron-inducible transcription of *ap65-1* through dual recognition and differential promoter selection toward both the MRE-1/MRE-2r and MRE-2f sites [118]. The TvMyb3 protein recognizes only the MRE-1 element, which activates basal and iron-inducible *ap65-1* transcription. Data demonstrated that TvMyb2 and TvMyb3 might coactivate basal and iron-inducible *ap65-1* transcription against TvMyb1 through conditional and competitive promoter entries [113,114,115,116,117,118]. An important part of the iron transcriptional regulatory mechanism is based on the phosphorylation and subsequent nuclear translocation of TvMyb3 in an iron concentration dependent-manner [116,117,118].

### 3.3. Posttranslational Regulation in T. vaginalis

Posttranslational regulation in *T. vaginalis*, which is mediated by protein-protein interactions, involves the participation of endogenous CP inhibitors called trichocystatins or posttranslational modifications (PTMs) modulated by different environmental signals including iron concentrations that may regulate the specific function of some trichomonad CPs and other parasite molecules.

Cystatins are endogenous CP inhibitors whose primary function is to protect the cell from undesired proteolysis [127]. Cystatins have been found in several species including nematodes, platyhelminths, bacteria, arthropods, and parasitic protists such as *Acanthamoeba* and *T. vaginalis* [122,127,128,129,130]. Although protist cystatins lack the characteristic domains necessary for CP inhibition activity in parasites, they are reversible CP inhibitors in addition to performing a wide variety of specific functions as part of parasite biology.

Three genes encoding trichocystatins (*tvicp-1*, *tvicp-2*, and *tvicp-3*), which are *T. vaginalis* cystatin-like endogenous CP inhibitors, have been identified in its genome [85]. Analysis of the *T. vaginalis* active degradome by mass spectrometry (MS) identified the trichocystatin-2 (TC-2) inhibitor together with TvCP39 in a 45 kDa protein spot [122]. TC-2 belongs to the stefin subfamily of the cystatin family I25, is located in the cytoplasm and lysosomes of the parasite and inhibits the proteolytic activity of papain, cathepsin L and some *T. vaginalis* cathepsin L-like CPs such as TvCP39 and TvCP65 [122].

TvCP39 is one of the CPs characterized as a *T. vaginalis* virulence factor that is localized on the parasite surface and that is involved in cytotoxicity to the target cell [17,86,98,99,105]. Recently, Puente-Rivera *et al.* (2015) [122] demonstrated the association between TvCP39 and TC-2. Both proteins colocalize in some cytoplasmic vesicles (possible lysosomes), suggesting that *in vivo* proteolytic activity regulation via protein-protein interactions may contribute to protecting the parasite from undesired excessive proteolytic activity [17,86,122]. Interestingly, TC-2 and TvCP39 association appears to be differentially modulated by iron (Puente-Rivera *et al.*, 2015, under revision). However, we could not rule out the possibility that the TC-2 inhibitor could also have a particular inhibitory function in the host cell CPs during the host-parasite interplay. Ongoing studies of the other two trichocystatins are being performed by our group. The presence of three endogenous inhibitors in *T. vaginalis* may suggest another level of regulation mediated by protein-protein interactions that may be modulated by iron and that may play key roles promoting parasitism by controlling and modulating the biological effects of CPs, including those described as virulence factors [86].

PTMs (glycosylation, phosphorylation, hypusination, ubiquitination, and others) play crucial roles in regulating the diverse protein-protein interactions that are involved in essentially all cellular process that are therefore required in every microorganism for its development. Thus far, PTM identification in *T. vaginalis* has been reported for only few proteins: TveIF5a, cytoskeletal proteins, tubulin, and several virulence factors, including P270, AP120, and TvCP39 [17,86,99,105,116,117,121].

*In silico* analysis of several *T. vaginalis* deduced amino acid sequences, including CPs, predicted putative *O*- or *N*-glycosylation and phosphorylation sites that were also suggested after *T. vaginalis* proteomic analysis [99,107]. Recently, after high-performance liquid chromatography and MS analysis Paschinger *et al.* (2012) [119] proposed that *T. vaginalis* has the machinery to carry on *O*- and *N*-glycosylation of proteins. The modification of *N*-glycans by *N*-acetygalactosamine in at least some strains is shared with the lipo(phospho)glycan and may represent a further interaction partner for host galectins. Thereby, PTMs may play key roles in the binding of parasites to host epithelial cells. Additionally, the variation in glycosylation among strains may be the result of genetic diversity within *T. vaginalis* [119]. Analysis of the *T. vaginalis* genome revealed that it has one of the largest eukaryotic kinomes, suggesting that trichomonads may perform protein phosphorylation reactions under different environmental conditions and through different signaling pathways [85]. Although the best studied PTM in *T. vaginalis* is protein phosphorylation, which has been demonstrated for TvLEGU-1 [96], *N*-glycosylation of TvCP39 has also been demonstrated [99]. However, we still do not know whether glycosylation is necessary for TvCP39 activation, whether glycosylation modulates its proteolytic activity, or its interaction with the endogenous inhibitor TC-2, or whether glycosylation is modulated by iron, which could help to explain changes in its molecular size as detected by MS [17,85,86,99,105,107,108].

Other unexplored mechanisms may be involved in expression regulation at the genomic and protein levels that may include regulation by microRNAs, epigenetic modifications, or repetitive elements. Additional information regarding other possible regulatory mechanisms has been recently reviewed in Arroyo *et al.* (2015) [17,86].

## 4. Posttranscriptional Regulation by Iron in *T. vaginalis*: A Regulatory System Parallel to the IRE-IRP System in *T. vaginalis*

A transcriptomic analysis of iron-regulated and iron-independent genes in *T. vaginalis* allowed the identification of 336 iron-regulated genes, of which 165 were up-regulated under high iron conditions and 171 under low iron conditions [112]. Thus far, the transcriptional iron regulatory elements have been identified and characterized only in the *ap65-1* gene, which is up-regulated by iron. The *ap65-1* gene has an iron-inducible core promoter and several regulatory elements that tightly control its expression in *T. vaginalis* [113,114,115,116,117,118] (Table 1). However, a sequence analysis of the 5' region of the other identified iron up-regulated genes reveals that none of them have an iron-inducible core promoter or the regulatory elements described in *ap65-1* and that they only share the presence of some MRE elements with *ap65-1*. Known regulatory sequences that may contribute to gene and protein expression depending on the iron concentration could not be identified in other genes. Thus, in *T. vaginalis*, some gene expression patterns under the influence of iron can be explained by the presence of a posttranscriptional regulatory mechanism.

### 4.1. Atypical IREs in T. vaginalis

As we previously mentioned herein, the posttranscriptional iron regulatory mechanism requires the interaction of two types of molecules: hairpin RNA structures (IREs) and cytoplasmic proteins (IRPs). Two atypical IRE-like hairpin-loop structures were identified and characterized in the trichomonad mRNAs of two CPs differentially regulated by iron that play key roles in trichomonal virulence, TvCP4 and TvCP12 (Table 1 and Table 2). The atypical hairpin structures, IRE-like *tvcp4* and IRE-like *tvcp12* have the characteristic elements of typical IREs: a terminal loop, a bulge, and upper and lower stems (Figure 2b,c). Although both trichomonad IRE-like structures are not completely similar to a consensus mammalian IRE structure (Figure 2a), both structures are able to bind the human IRP in *in vitro* REMSA assays. Functional data using cytoplasmic extracts from *T. vaginalis* grown under low iron conditions also suggest the presence of a parallel iron posttranscriptional regulatory mechanism mediated by atypical hairpin RNA structures that bind to atypical multifunctional cytoplasmic proteins in *T. vaginalis*, an early-branching amitochondriate parasite [17,60,61].

#### 4.1.1. IRE-Like tvcp4

**Figure 4:**
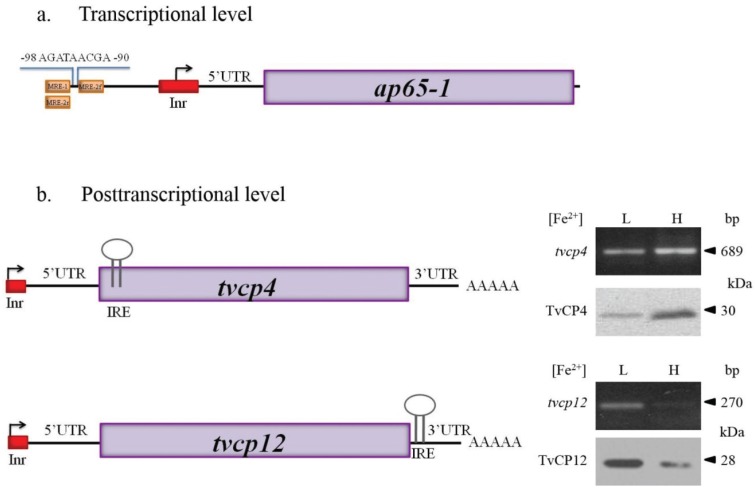
Transcriptional and putative posttranscriptional gene expression regulation by iron in *Trichomonas vaginalis*. (**a**) Transcriptional regulation. Top panel shows the iron-responsive promoter elements of the *ap65-1* gene. The graphic shows the initiator element, Inr (red box), the sequence of the iron-responsive core promoter, and the Myb recognition element (MRE) domains (light orange boxes); (**b**) Posttranscriptional regulation. The bottom panel shows the genomic organization and IRE-like localization in *tvcp4* and *tvcp12* mRNAs. Diagrams also illustrate the Inr elements located at the *tvcp4* 5'-region, the position of the stem-loop structure (IRE-like) and *tvcp4* expression and amounts of protein. The *tvcp4* gene has an IRE-like structure at the 5' coding region that appears to affect mRNA translation without major changes in the amount and stability of the mRNA. This effect could be due to the interaction between the IRE-like *tvcp4* structure and cytoplasmic RNA-binding proteins in the absence of iron that prevents mRNA translation. When the IRE is located at the 3'-UTR, as in the *tvcp12* mRNA, its stability increases possible due to RNA-protein interactions. Thus, more protein is observed under low iron concentrations than under high iron concentrations ([61], León-Sicairos *et al.*, 2015 under revision).

IRE-like *tvcp4* is located at the 5' region of the *tvcp4* mRNA. TvCP4 is an iron up-regulated cysteine proteinase involved in hemolysis (Figure 4) [60,61,101]. RT-PCR analysis using RNA from parasites grown under different iron concentrations showed no major changes in the *tvcp4* mRNA levels under different iron concentrations. However, Western blot analysis showed the presence of a greater amount of TvCP4 under high than under low iron concentration as expected for a translational blockage in the absence of iron when the IRE is located at the 5' region (Figure 4b). As shown in Figure 2, the IRE-like *tvcp4* hairpin structure was classified as a G_1_C5-type IRE-like structure that possesses a bulge with an A at the 3'-strand, similar to the previously described IRE-like structure found in the 75 kDa subunit of mitochondrial complex I [61,68]. Torres-Romero and Arroyo (2009) [61] also found that the IRE-like *tvcp4* contains a conserved C nucleotide, five bases upstream from the loop sequence at the 5'-strand, similar to the C-type IREs. Phylogenetic analysis using the nucleotide sequence of IRE-like *tvcp4* (6-nt loop GGCACA) compared with the previously reported mammalian elements derived from IRE-*fer* and IRE-like motif SERTAD2 [24,131], PfIRE-3 [77], and the bacterial IRE-like *gerE* from *Bacillus subtilis* [132] revealed a relationship between IRE-like *tvcp4* and IRE-like *gerE* and the IRE-like motif SERTAD2. A structure comparison using the Mfold program showed an atypical “hexaloop” and five-paired nucleotides in the upper stem of the hairpin in IRE-like *tvcp4*. The comparative analysis also revealed that IRE-like *tvcp4* is phylogenetically more related with bacterial structures than with the other IRE sequences analyzed [61]. REMSA data showed that the IRE-like *tvcp4* structure interacted with recombinant human IRP and trichomonad cytoplasmic proteins from parasites grown under low iron concentrations [60,120].

#### 4.1.2. IRE-Like *tvcp12*

IRE-like *tvcp12* predicted secondary structure revealed an apical tetraloop that differed from the consensus structure but that was similar to the functional IRE-*fer* mutants and the human *AHSP* mRNA [54,61,133]. Taking these data into account, Torres-Romero and Arroyo (2009) [61] considered the U-G base pair next to the loop as part of the 6-nt sequence (UAAUUG) involved in IRP binding. Phylogenetic analysis revealed a possible relation with the plasmodial IRE, suggesting the presence of protozoan-specific structures. IRE-like *tvcp12* contains a conserved C five bases upstream from the loop sequence at the 5'-strand, similar to the C-type IREs; however, instead of the C nucleotide forming a bulge, it possesses a U-bulge intra-loop ([49]; León-Sicairos *et al.*, 2015 under revision). An identical loop was found when IRE-like *tvcp12* was compared with the loop of the 3' (+) 42 of the MHV genome, which is capable of binding mitochondrial aconitase [134,135]. TvCP12 is over-expressed under low iron conditions (Figure 4b) and is involved in *T. vaginalis* cytotoxicity (our unpublished data; León-Sicairos *et al.*, 2015 under revision). As shown in Figure 4, increases in mRNA and protein amounts were observed under low iron conditions, as expected for mRNA stabilization due to the location of an IRE hairpin at the 3′-UTR and its interaction with cytoplasmic RNA-binding proteins. Functional analysis of IRE-like *tvcp12* demonstrated the capability of the IRE-like *tvcp12* hairpin to stabilize the mRNA half-life (León-Sicairos *et al.*, 2015 under revision).

To study the iron-regulated posttranscriptional mechanism in *T. vaginalis,* we performed REMSAs to demonstrate that the atypical hairpin structures identified in trichomonad CP mRNAs IRE-like *tvcp4* and IRE-like *tvcp12* are functional; we used IRE-*fer* as a control. The tested hairpin structures specifically interacted with the human recombinant protein IRP-1 (hIRP-1r) and cytoplasmic proteins from HeLa cells and from trichomonads grown under iron-restricted conditions ([60,120]; León-Sicairos *et al.*, 2015 under revision). Our next approach was to identify the cytoplasmic RNA-binding proteins in *T. vaginalis* that interact with the tested hairpin structures*.*

### 4.2. RNA-Binding Proteins in T. vaginalis

As we discussed initially, posttranscriptional mechanisms for controlling protein synthesis are generally mediated by specific RNA-protein interactions. To perform a complete characterization of the iron-regulated posttranscriptional mechanism in *T. vaginalis*, the RNA-binding proteins implicated in the interaction with IRE hairpin RNA structures had to be identified because *T. vaginalis* does not have the Krebs citric acid cycle or aconitase activity [85,90,136,137].

*In silico* analysis of the *T. vaginalis* genome and experimental data revealed the absence of genes encoding for IRP, IRP-like proteins, or aconitases in trichomonads [85,90,136,137]. However, experimental data obtained by crosslinking, REMSA competition, and Northwestern blot (NWB) assays using radiolabeled IRE-like *tvcp4* and IRE-*fer* RNA probes and cytoplasmic extracts from parasites grown under iron-restricted conditions, suggested the presence of a parallel IRE/IRP system in *T. vaginalis* ([60,120]; León-Sicairos *et al.*, 2015 under revision). Mass spectrometry (MS) also helped to identify cytoplasmic proteins with RNA-binding ability that specifically interacted with IRE-like *tvcp4*, forming specific RNA-protein complexes (RPCs) [120]. The addition of a molar excess of unlabeled homologous or heterologous IRE-like probes or unrelated RNA demonstrated the specificity of the RPCs formed between trichomonad IRE-like hairpins and RNA-binding proteins in *T. vaginalis*. Subsequent studies with IRE-like *tvcp12* also demonstrated that this trichomonad hairpin structure binds and competes with the other IRE structures (IRE-like *tvcp4* and IRE-*fer*) for the same RNA-binding proteins (León-Sicairos *et al.*, 2015 under revision).

**Figure 5:**
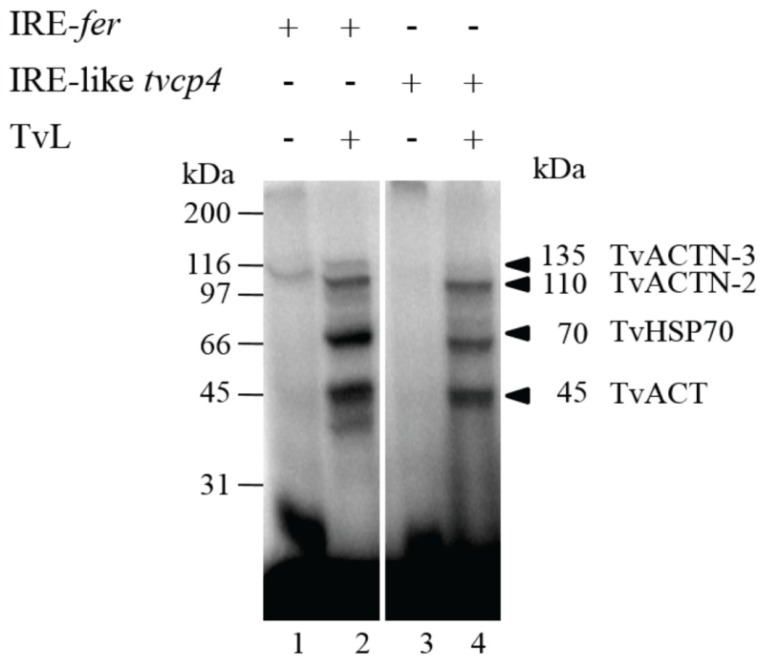
Identification of *T. vaginalis* RNA-binding proteins by UV crosslinking assays using different IREs as probes and cytoplasmic extracts from parasites grown under iron-restricted conditions. UV crosslinking assay of ^32^P-labeled IRE-*fer* and, IRE-like *tvcp4* with cytoplasmic extracts from *T. vaginalis* grown under iron-restricted conditions (TvL). Molecular markers are indicated in kilodaltons (kDa). The arrowheads indicate the position of the RNA-protein complex bands of 135, 110, 70 and 45 kDa.

Crosslinking and NWB assays revealed that both *T. vaginalis* IRE-like RNAs and the mammalian IRE-*fer* interacted with the same trichomonad cytoplasmic proteins, but with different intensity. Proteins of 135, 110, 70, and 45 kDa were identified by MS and their molecular characterization allowed determination of their function as RNA-binding proteins (Figure 5). The MS identification showed that the 135 kDa protein corresponds to α-Actinin-3 (TvACTN3); the 110 kDa protein, to α-Actinin-2 (TvACTN2); the 70 kDa protein, to the cytoplasmic Heat Shock Protein 70 (TvHSP70); and the 45 kDa protein, to actin (TvACT). Although the molecular characterization as RNA-binding proteins that interact with trichomonad IREs has not yet been established for TvACTN-2 and TvACT, we propose that all four proteins are integrated in a multiprotein complex that interact with *T. vaginalis* IREs under iron-restricted conditions ([17,60,120]; León-Sicairos *et al.*, 2015 under revision).

**Figure 6:**
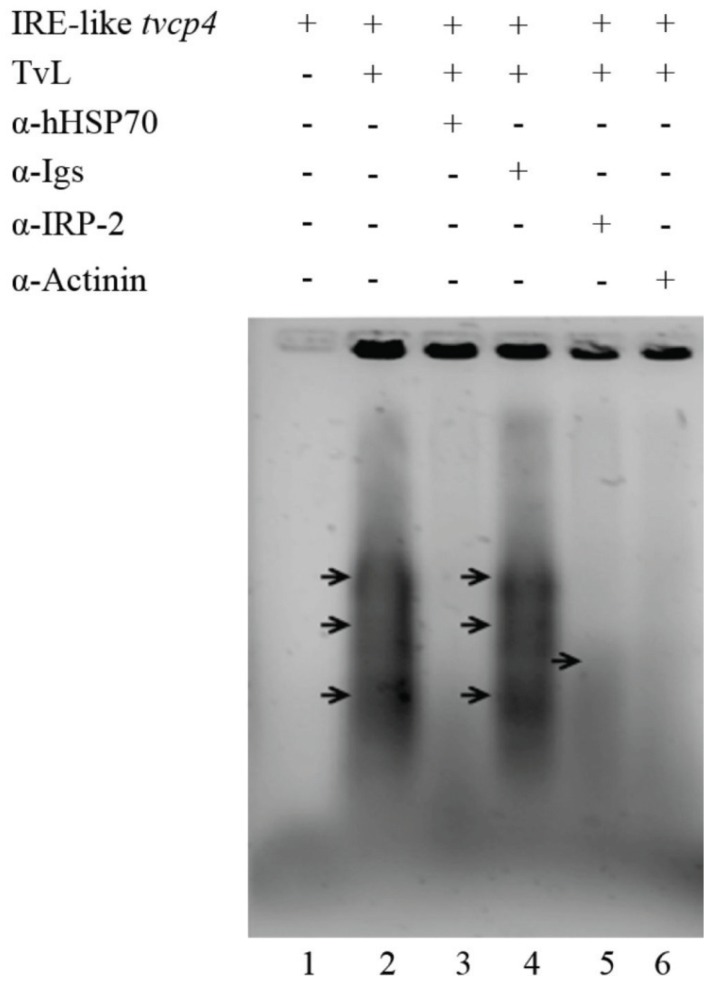
Identification of RNA-binding proteins in the RNA-protein complexes formed between IRE-like *tvcp4* and *T. vaginalis* cytoplasmic extracts. Non-radioactive supershift RNA-protein gel shifting assay (REMSA) with specific antibodies confirmed the interaction of IRE-like *tvcp4* with cytoplasmic proteins from parasites grown under iron-restricted conditions (TvL) in the presence of antibodies directed against hHSP70, α-Actinin and IRP-2, and human immunoglobulins (α-Igs). The last two antibodies were used as positive and negative controls, respectively. Arrows point to the RNA-protein complexes that disappear in the presence of specific antibodies.

To corroborate the interaction of trichomonad proteins with IRE-like *tvcp4*, we performed a supershift REMSA using heterologous antibodies against individual proteins. As shown in Figure 6, the addition of specific antibodies caused the disappearance of the RNA-protein complexes, indicating the presence of HSP70, actinin, and a protein that cross-reacted with IRP-2 in the RNA-protein complexes.

Interestingly, all the proteins identified that are part of the RNA-multiprotein complex that interact with the *T. vaginalis* IREs have typical functions, as cytoskeleton-related proteins or as stress response proteins. Data suggest that actinin, actin, and HSP70 are another group of multifunctional or moonlighting proteins of *T. vaginalis* with alternative functions as RNA-binding proteins that interact with RNA hairpin structures and that may participate in iron-mediated posttranscriptional regulation in *T. vaginalis*. In the subsequent sections, we will address each of the proteins to show evidence demonstrating their function as RNA-binding proteins.

#### 4.2.1. TvHSP70 as an RNA-Binding Protein in *T. vaginalis*

The 70 kDa Heat Shock protein (HSP70) is a ubiquitous protein highly conserved throughout evolution. The HSP70 family includes highly conserved molecular chaperones that are present in most subcellular compartments of eukaryotic cells. HSP70, together with the J domain of co-chaperones and nucleotide exchange factors, assists in a multitude of different cellular protein-folding processes during protein maturation [138]. HSP70 assists in the assembly and folding of newly synthesized proteins, refolding of misfolded or aggregated proteins, membrane translocation of organellar and secreted proteins, and the control of regulatory proteins [139]. This ATP-dependent chaperone protein has high versatility to fold different unfolded proteins. Its folding capacity is due to its ability to recognize a short degenerated motif of five primarily hydrophobic amino acid residues that is found approximately every 30–40 residues in almost all proteins and that is only exposed when the protein is misfolded [140].

**Figure 7:**
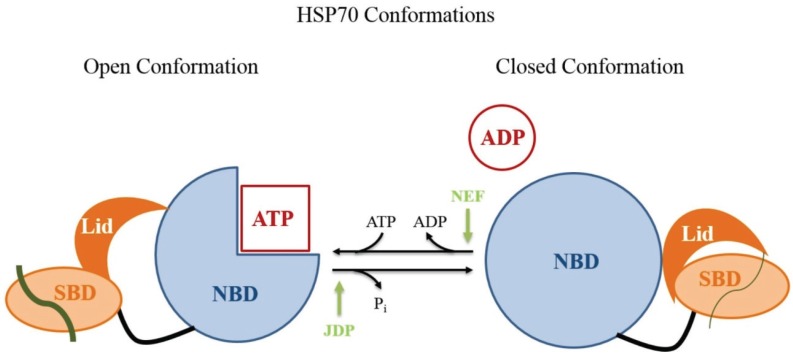
HSP70 open and closed conformations. Open conformation: the nucleotide-binding domain (NBD) is shown in light blue. When bound to ATP, this domain allows the peptide to associate with the substrate-binding domain (SBD) shown in orange. The SBD presents a region that acts as a lid (dark orange). In the HSP70 open conformation, the association and dissociation of peptides (gray lines) with the SBD occur at high rates. The essential process of ATP hydrolysis is stimulated by the co-chaperone J-domain protein (JDP) and by nucleotide exchange by nucleotide exchange factors (NEFs). Closed conformation: after ATP hydrolysis, peptide association and dissociation decreases by several orders of magnitude, which leads to an increase in the affinity for substrate peptides, suggesting allosteric control by the lid (Figure modified from Mayer, 2013 [144]).

HSP70 is formed by an N-terminal nucleotide-binding domain (NBD) and a C-terminal polypeptide substrate-binding domain (SBD). HSP70 has two conformations in the SBD: open and closed (Figure 7). The SBD contains the peptide-binding pocket and an α-helical subdomain that acts as a lid [141]. The NBD consists of four α-β subdomains divided into two lobes by a central ATP-binding cleft [142]. The SBD and the NBD are connected via a flexible highly conserved linker [143]. The affinity of HSP70 for polypeptides is regulated by the NBD. When ATP is bound, the association and dissociation between the peptide and the SBD occur at high rates, resulting in a rather low affinity for polypeptides. In the second state, after ATP hydrolysis by the NBD, the peptide association and dissociation rates decrease, leading to an increase in the affinity for polypeptides [144].

Recently, biochemical and proteomic analyses of stress-induced chaperone HSP70 and the constitutive chaperone Hsc70 revealed the importance of ubiquitination to maintain the balance of chaperones in the cell [145]. When these chaperones do not have protein bound, the heat shock protein co-chaperone CHIP performs HSP70 and Hsc70 polyubiquitination in several Lys residues spread throughout the protein. A detailed comparison between the Lys residues ubiquitinated in both proteins revealed that 12 of 39 Lys residues in HSP70 protein and 16 of 45 in Hcs70 were ubiquitinated. Differences in the ubiquitinated residues may provide clues to explain how HSP70 and Hcs70 chaperones are degraded at different rates in the proteasome [145].

In *T. vaginalis*, we found a cytoplasmic TvHSP70 protein as part of the RPC that interacts with IRE-like *tvcp4* and IRE-like *tvcp12* that may regulate the differential expression of TvCP4 and TvCP12 by iron. *In silico* analysis of the *T. vaginalis* genome showed the presence of eleven *tvhsp70* genes with high sequence identity among them ([85]; www.trichdb.org) (Table 3). To determine which TvHSP70 is an RNA-binding protein involved in RPC formation, we separated cytoplasmic protein extracts from parasites grown under iron-restricted conditions by 2-DE and performed NWB assays with IRE-like *tvcp4* as a probe and Western blot analysis using an antibody directed against hHSP70. A spot of ~70 kDa was detected by NWB and was recognized by the anti-hHSP70 antibody. MS analysis identified the 70 kDa spot as a specific cytoplasmic TvHSP70 with the ID TVAG_044510 (*tvhsp70-4*) (www.trichdb.org) (Arroyo *et al.*, 2015 under revision).

**Table 3:** Expressed sequence tag (EST) analysis of *tvhsp70*, *tvactn,* and *tvact* encoding genes expressed in *T. vaginalis* under different environmental conditions found in the *T. vaginalis* genome sequence (www.trichdb.org).

ID	Name	Total EST ^a^	Identity (%)
TVAG_044510	HSP70-4 *	66	100
TVAG_161100	HSP-70 putative	16	96.52
TVAG_151220	HSP70-4	22	96.16
TVAG_163000	HSP putative	17	96.00
TVAG_137250	HSP70-4	5	95.44
TVAG_206270	HSP putative	14	95.80
TVAG_300470	HSP putative	14	95.24
TVAG_184320	HSP putative	12	95.39
TVAG_171670	HSP putative	10	97.17
TVAG_092490	HSP-70 putative	104	62.80
TVAG_340390	HSP70-4	25	57.06
**ID**	**Name**	**Total EST ^a^**	**Identity (%)**
TVAG_156680	α-Actinin-1	112	51.66
TVAG_190450	α-Actinin-2	1086	61.96
TVAG_239310	α-Actinin-3*	495	100
TVAG_247460	α-Actinin-4	2	41.92
TVAG_260390	α-Actinin-5	1	41.82
**ID**	**Name**	**Total EST ^a^**	**Identity (%)**
TVAG_054030	actin putative *	338	100
TVAG_090470	actin putative	231	99.12
TVAG_149090	actin putative	141	98.67
TVAG_150270	actin putative	140	90.80
TVAG_160060	actin putative	168	99.20
TVAG_172680	actin putative	503	98.67
TVAG_200190	actin putative	322	99.12
TVAG_247170	actin putative	3	56.30
TVAG_249200	actin putative	404	98.85
TVAG_310030	actin putative	239	98.50
TVAG_337240	actin putative	181	98.50
TVAG_485210	actin putative	73	98.67
TVAG_512800	actin putative	86	99.17
TVAG_534990	actin putative	75	99.30

The genomic identification number (ID), the common name, total ESTs are indicated and the percent of identity (%) among genes from the same family is indicated. * The percent of identity is based on the sequence that encodes for each RNA-binding protein identified in our work. ^a^ The total EST corresponds to those reported in www.trichdb.org database from libraries of parasites under different environmental conditions: trichomonads at mid-log, HMW C1, Cot6 normalized, with amoeboid form, fibronectin-mediated adherence, in log-phase; arrested in G2/M, under cold stress, under low iron conditions, under low glucose conditions; and adherence to VEC.

**Figure 8:**
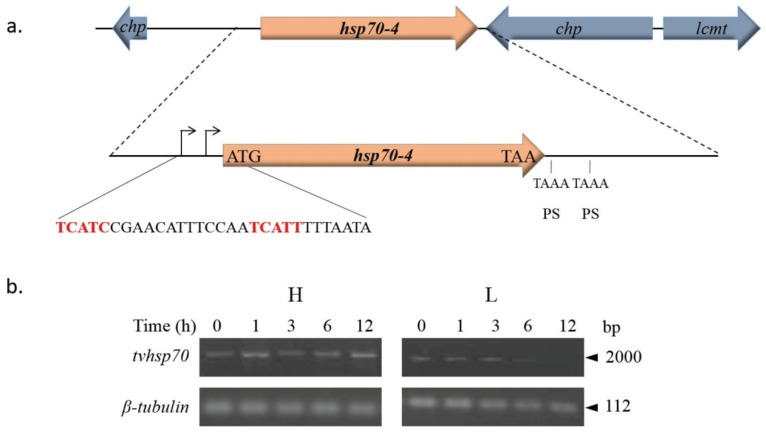
Genomic organization and analysis of *tvhsp70-4* mRNA stability. (**a**) Genomic organization of the *tvhsp70-4* gene TVAG_044510. The positions of the *tvhsp70-4* gene and the surrounding genes in the contig DS113536 are shown. *chp*: conserved hypothetical protein; *lcmt*: leucine carboxyl methyltransferase encoding genes. An amplification of the sequence surrounding the *tvhsp70-4* gene is also shown. The 5'- and 3'-UTRs predicted from the *T. vaginalis* genome sequence [85]. The 5'-UTR contains two putative *T. vaginalis* initiator (Inr) elements (curved arrow, red sequence). Initiation (ATG) and stop (TAA) codons are also indicated. The 3'-UTR has two putative polyadenylation signals (PSs), TAAA, downstream of the stop codon. Other previously described elements necessary for mRNA polyadenylation were not found [146]; (**b**) Agarose gels show the *tvhsp70-4* amplicons after RT-PCR assays of RNA from actinomycin D-treated parasites grown under different iron conditions and harvested at different times (0, 1, 3, 6, and 12 h) after transcriptional blockage. The *β-tubulin* gene was used as a loading control. L: low or H: high iron conditions.

*In silico* analysis of the TVAG_044510 *tvhsp70-4* gene and its regulatory sequences showed the presence of two putative Inr elements containing putative TSSs. At the 3'-UTR, *tvhsp70-4* contains two possible polyadenylation signals but no other regulatory elements as previously reported by Espinosa *et al.* (2002) [146] (Figure 8a).

Considering the differences in the 3'-UTR, we performed a transcriptional blockage with actinomycin D to evaluate the influence of iron on the stability of this mRNA and to determine whether the differences detected in the 3'-UTR could affect TvHSP70 expression. Figure 8b shows that *tvhsp70-4* has higher expression under high than under low iron conditions. However, no apparent major differences were observed in terms of mRNA stability. Considering the high percentage of identity among the *tvhsp70* genes in this organism (Table 3), we may be observing the global iron effect over all the *tvhsp70* genes expressed under different iron conditions (not a particular gene). Thus, to determine the possible differential expression levels among the distinct *tvhsp70* genes of *T. vaginalis*, we analyzed the reported ESTs in the *T. vaginalis* genome database and the transcriptomic and proteomics data reported thus far.

Global EST analysis revealed that *tvhsp70-4* (TVAG_044510) and *tvhsp70-putative* (TVAG_092490) have more transcripts than other *tvhsp70* genes (Table 3). The transcriptomic analysis performed by Horváthová *et al.* (2012) [112] showed the expression of only four *tvhsp70* genes under high iron conditions that corresponded to the ID numbers TVAG_044510, TVAG_206270, TVAG_092490, and TVAG_340390. The *tvhsp70-4* gene (TVAG_044510), whose product was identified as an RNA-binding protein (Arroyo *et al.*, 2015 under revision) showed 11-fold induction under high iron conditions [112]. Moreover, in the proteomic analysis performed by de Jesus *et al.* (2007) [107], the peptides identified under low or high iron conditions are the most conserved among TvHSP70s, but a particular TvHSP70 protein could not be distinguished [107].

The principal differences in TvHSP70 expression were observed by 2-DE-WB using antibody directed against the recombinant TvHSP70-4r and indicated that proteins from parasites grown under iron-rich conditions were degraded by an unknown proteolytic activity (Arroyo *et al.*, 2015, under revision). TvHSP70-4 degradation could be through the proteosomal system similar to that for IRP-2 under iron-rich conditions by a mechanism that could involve ubiquitination of TvHSP70-4 by an E3 ubiquitin ligase-like protein [31,32]. Future studies are necessary to determine whether the ubiquitination process is involved in TvHSP70-4 degradation under iron-rich concentrations as a key element in a parallel IRE-IRP-like mechanism in *T. vaginalis*. Work is in progress to solve these questions.

In immunolocalization assays with the anti-TvHSP70-4r antibody and parasites grown under different iron conditions, the TvHSP70-4 cytoplasmic fluorescence intensity appeared weaker in the presence of iron as compared with the strong fluorescence of parasites grown under low iron conditions. In low-iron parasites the fluorescence signal was also observed close to the nucleus; probably in the endoplasmic reticulum where protein translation occurs. Differences in TvHSP70-4 fluorescence intensity depending on the iron conditions are in agreement with the 2-DE-WB results, suggesting that less TvHSP70-4 protein in the presence of iron could be related to greater degradation rates than in the absence of iron (Arroyo *et al.*, 2015 under revision).

The ability of TvHSP70-4 to specifically bind to *T. vaginalis* RNA hairpin structures in the absence of iron was demonstrated by supershift assays adding anti-TvHSP70-4r antibody to the REMSA samples. A reduction in the RPC radioactive signal formed between the cytoplasmic extracts of low iron *T. vaginalis* proteins and the trichomonad IRE-like *tvcp4* RNA probe was observed in the presence of anti-TvHSP70-4r antibody. Similar results were obtained when IRE-*fer* or the IRE-like *tvcp12* RNA probes were used instead. Thus, data indicate that TvHSP70-4 is one of the RNA-binding proteins that bind to trichomonad IRE-like hairpins (Arroyo *et al.*, 2015 under revision; León-Sicairos *et al.*, 2015 under revision).

The previous results were corroborated using purified TvHSP70-4r in a NWB assay that included IRE-like *tvcp4*, an IRE-like *tvcp4* deletion mutant, or an unrelated RNA structure as probes. The data demonstrate that TvHSP70-4r specifically interacted with the trichomonad IRE-like *tvcp4* probe and that the IRE-like *tvcp4* mutant was incapable of binding to TvHSP70-4r, confirming that TvHSP70-4r can function, at least *in vitro*, as an RNA-binding protein that binds the atypical trichomonad IRE mRNA structures (Arroyo *et al.*, 2015 under revision). Similar results were obtained using IRE-like *tvcp12* and its mutant (León-Sicairos *et al.*, 2015, under revision).

Previous studies have shown that the N-terminal ATP-binding domain of hHSP70 represents an RNA-binding domain modulated *in vitro* by ATP and ATP hydrolysis is essential for HSP70 chaperone activity [147,148]. REMSAs with IRE-like *tvcp4* and purified TvHSP70-4r in the presence of increasing concentrations of ATP (0.5–5.0 mM) were used to examine the capacity of ATP to modulate the RNA-binding activity of TvHSP70-4r. These experiments revealed that the IRE-binding capability of TvHSP70-4r was reduced by the presence of ATP in a concentration-dependent manner. Data strongly suggest that the ATP-binding domain of TvHSP70-4r may be involved in the specific IRE-binding activity and could function as the RNA-binding domain of TvHSP70-4r to the IRE-like *tvcp4* structure of *T. vaginalis* in the absence of ATP (Arroyo *et al.*, 2015, under review). Thus, data also suggest that TvHSP70-4 could function as an alternative RNA-binding protein involved in the posttranscriptional regulation mediated by iron in *T. vaginalis* in the absence of IRPs and aconitases.

#### 4.2.2. TvACTN3 and TvACTN2 as RNA-Binding Proteins in *T. vaginalis*

α-Actinin is a cytoskeletal actin-binding protein that belongs to the spectrin subfamily. The spectrin subfamily is characterized by the presence of a specific number of spectrin repeats. Other known α-actinin functions are related to the association of the cytoskeleton to different transmembrane proteins and regulation of the activity of several receptors, serving as a scaffold to connect various signaling pathways in the cytoskeleton. During evolution, gene duplication generated a wide diversity of α-actinins. In mammals α-actinin diversity is very pronounced; four genes produce at least six α-actinin products or isoforms, and each isoform has different subcellular and tissue localization [149].

In eukaryotes, α-actinins contain four spectrin repeats that are assembled as rod-shape homodimers with a subunit molecular mass of 94–103 kDa [150]. The actin-binding site is located at the *N*-terminal and contains two calponin-like domains, where the closest *N*-terminal domain binds actin [151]. Two EF-hand domains, important for calcium binding, are located at the *C*-terminal [152]. The α-actinin *N*- and *C*-terminal domains are connected through the rod domain, which is formed by spectrin repeats; each repeat contains ~106 amino acid residues [153]. The rod domain frequently presents four spectrin repeats and is important for α-actinin dimerization. The distance of the rod domain determines the distance between cross-linked actin filaments and serves as both an interaction site for receptors and an adaptor protein. The number of spectrin repeats has implications in the cytoskeleton organization [153].

Five annotated sequences in the *T. vaginalis* genome correspond to α-actinin genes: TVAG_156680 (*tvactn1*), TVAG_190450 (*tvactn2*), TVAG_239310 (*tvactn3*), TVAG_247460 (*tvactn4*) and TVAG_260390 (*tvactn5*) [85]. Using SMART, MOTIF SCAN, and PROSITE programs, their deduced amino acid sequences were analyzed to identify the characteristic α-actinin domains. All actinins have two calponin domains in the *N*-terminal region that confer the actin-binding capacity in other organism. Moreover, in the central region the location of the spectrin repeats or rod domain is associated with the protein structure. Finally, two EF-hand domains are present in the *C*-terminal region. *T. vaginalis* α-actinins have different molecular weights due to the number of spectrin repeats present in each α-actinin and the presence of specific motifs in the central domain of TvACTN4 and TvACTN5 [120]. Iron-mediated expression of the five *T. vaginalis* α-actinin genes was analyzed by qRT-PCR. The *tvactn1*, *tvactn2*, and *tvactn3* genes are over-expressed under high iron conditions, while *tvactn4* and *tvactn5* are not expressed in any of the studied iron conditions [120]. Our qRT-PCR results are consistent with the EST analysis of *tvactn* gene expression in the *T. vaginalis* genome database (www.trichdb.org) (Table 3).

Crosslinking assays revealed the presence of 135 and 110 kDa proteins in the ribonuclear protein complexes formed between *T. vaginalis* cytoplasmic extracts from parasites grown under low iron conditions and IRE-like *tvcp4* and IRE-like *tvcp12* RNA probes (Figure 5 and data not shown) ([120]; León-Sicairos *et al.*, 2015 under revision). The MS analysis demonstrated that these proteins correspond to TvACTN3 (135 kDa; TVAG_239310) and TvACTN2 (110 kDa; TVAG_190450).

To confirm that TvACTN3 is one of the proteins involved in the interaction with IRE-like *tvcp4* RNA, the *tvactn3* gene was cloned to produce the recombinant protein, TvACTN3r, and polyclonal antibodies directed against TvACTN3r were produced. TvACTN3 contains three functional domains: domain I, which includes an actin-binding domain or calponin domain (*N*-terminal region); domain II, which contains four spectrin repeats; and domain III, which contains two EF-hand domains. The three functional domains were also cloned, expressed as recombinant proteins and used for functional assays. We performed functional assays to determine whether TvACTN3 is another RNA-binding protein of *T. vaginalis* that may participate in the iron-regulated posttranscriptional mechanism in trichomonads. Supershift, NWB, and cross-linking results revealed that TvACTN3 specifically interacts with the IRE-like *tvcp4* RNA probe through its domain II, suggesting that TvACTN3 functions as an RNA-binding protein [120]. A sequence analysis of TvACTN-3-Domain II at the protein level using the SMART program identified several putative RNA-binding motifs: BRIGHT/ARID (DNA binding motif), B5 (RNA-binding motif with the capacity to bind magnesium and ATP), LA, KH, and a Pumilio domain.

**Figure 9:**
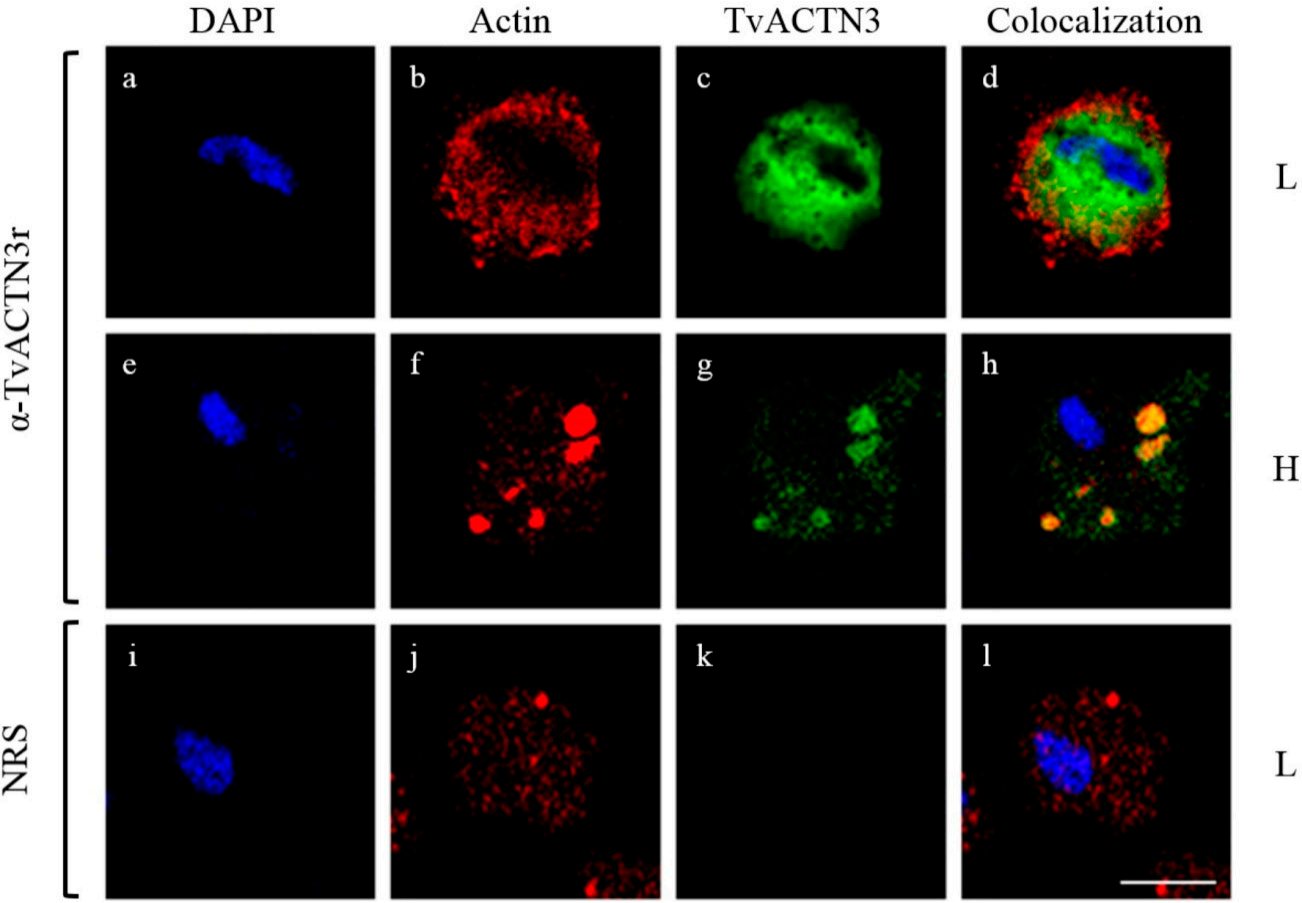
Cytoplasmic localization of TvACTN3 as determined by indirect immunofluorescence and confocal microscopy. L: Parasites grown under low iron conditions (panels a–d; i–l) or H: under high iron conditions (panels e–h) were incubated with antibody directed against TvACTN3r (panels c, g, k) and with secondary antibody goat anti-rabbit-FITC. As a negative control, parasites grown under low iron conditions were incubated with pre-immune serum (NRS) (panels i–l). Samples were analyzed by confocal microscopy. DAPI (nucleus, blue); phalloidin-rhodamine (actin, red) and FITC-fluorescence (actinin, green). Scale bar in l represents 5 µm.

TvACTN3 localizes to the cytoplasm under both high and low iron concentrations as expected for an RNA-binding protein that may participate in a posttranscriptional regulatory mechanism [120]. Its cytoplasmic localization was confirmed by indirect immunofluorescence and confocal microscopy assays using anti-TvACTN3r antibody and phalloidin to detect filamentous actin under different iron concentrations. As shown in Figure 9, TvACTN3 and actin localize in the cytoplasm, but their distribution change depending on the iron concentration (Figure 9, panels b, c, f, g). The distribution and association of TvACTN3 with filamentous actin changed according to the iron conditions. TvACTN3 and actin are dispersed throughout the cytoplasm in parasites grown under low iron conditions (Figure 9, panels b–d). Under high iron conditions, both proteins partially co-localized in the cytoplasm in aggregates and actin clusters (Figure 9, panels f–h), and no signal is detected in the nucleus (Figure 9, panels a and e). The pre-immune serum used as a negative control showed no TvACTN3 or actin signal, as expected (Figure 9, panel k). The perinuclear TvACTN3 signal is also observed when the parasites are grown under iron-depleted concentrations and this signal partially colocalized with actin (data not shown). Data suggest that TvACTN3 and actin molecules associate primarily under high iron conditions and that TvACTN3 functions as an actin-binding protein (see below). In contrast, under low iron conditions, TvACTN3 functions as an RNA-binding protein that may participate in the iron-regulated posttranscriptional mechanism of *T. vaginalis*.

The 110 kDa actinin protein TvACTN2 was also found in the RPC formed by cytoplasmic proteins from parasites grown under low iron conditions that interacted with *T. vaginalis* IRE-like *tvcp4* and IRE-like *tvcp12* RNA probes ([120]; León-Sicairos *et al.*, 2015, under revision). TvACTN2 was previously reported in cytoplasm of ovoid forms of *T. vaginalis*. In amoeboid forms of *T. vaginalis*, a high concentration of TvACTN2 is present in the cell periphery, primarily in pseudopods and adhesion plates, and colocalizes with actin [154]. TvACTN2 is over-expressed during adherence and under low iron conditions (Table 3, [112,120]). Additional experiments are necessary to confirm the participation of TvACTN2 in the RPC that interacts with the *T. vaginalis* IRE-like structures.

A molecular evolution study with α-actinins from different species, including the *T. vaginalis* α-actinin TvACTN2, revealed that only the first of the five spectrin repeats shows some similarity with α-actinins of other organisms, suggesting that the other four repeats have evolved possible due to intragenic duplication that has occurred in other sequences [155,156]. The study also suggested alternative functions for TvACTN2.

#### 4.2.3. TvACT as an RNA-Binding Protein in *T. vaginalis*

Actin filaments form cross-linked networks that enable eukaryotic cells to transport cargo, change shape, and move. Other potential functions and biochemical targets have been identified. Nuclear actin has been implicated in transcription by all three RNA polymerases and is an important component of chromatic regulatory complexes [157,158].

Actin is one of the most abundant proteins of *T. vaginalis*; TvACT constitutes about 10% of the total proteins of *T. vaginalis*, and it is primarily concentrated in the cortical region where the pseudopodia are formed [159]. TvACT displays biochemical and biophysical properties that differ from those of muscle actin. In the genome of *T. vaginalis* many genes encoding for proteins that interact with actin have been described; however, only some of these genes have been experimentally demonstrated. Coronin has been shown to attach to the existing polymerized actin (F-actin) in the parasite during actin polymerization [160].

**Figure 10:**
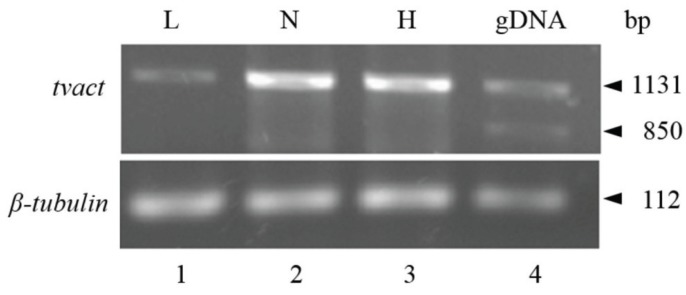
Differential *tvact* gene expression depending on the iron concentrations. *tvact* RT-PCR assays of RNA extracted from parasites grown under different iron conditions. β*-tubulin* gene expression was used as a loading control. L: low iron conditions; N: normal iron conditions; H: high iron conditions; gDNA, genomic DNA used as a template control PCR.

We observed an interaction with a 45 kDa band in a crosslinking assay using cytoplasmic extracts from parasites grown under low iron conditions and radiolabeled IRE-like *tvcp4* and IRE-like *tvcp12* RNA probes (Figure 5). MS analysis of the 45 kDa spots detected by 2-D-NWB assays identified two *T. vaginalis* actin proteins, TVAG_054030 and TVAG_200190, suggesting the involvement of actins as other RNA-binding proteins that interact with the trichomonad hairpin structures. Interestingly, twelve *tvact* genes and eleven actin-related genes are present in the *T. vaginalis* genome sequence [85] (Table 3). Alignment of the *tvact* gene sequences showed a high percentage of identity among them that is also observed in the amino acid sequences (Table 3). EST analysis of all *tvact* sequences shows that genes with the highest expression corresponded to TVAG_172680, TVAG_249200, TVAG_054030, and TVAG_200190 (Table 3). Two *tvact* genes correspond to the TvACT proteins identified by 2-D-NWB and MS analysis. RT-PCR analysis with mRNA extracted from *T. vaginalis* parasites grown under different iron concentration reveals that the *tvact* genes are up-regulated by iron (Figure 10).

Another important result that helped us to understand the possible participation of actin as part of the RPC that forms between cytoplasmic proteins and the IRE-like *tvcp4* RNA was provided in the immunofluorescence colocalization analysis using phalloidine rodamine and antibodies directed against TvACTN3r in parasites grown under different iron concentrations. Figure 9 shows colocalization between TvACTN3 and filamentous actin only in parasites grown under high iron conditions. However, additional experiments need to be performed to establish the relevance of actin in the *T. vaginalis* posttranscriptional mechanism mediated by iron. Thus, work is in progress to achieve this aim.

## 5. A Posttranscriptional Iron Regulatory Mechanism in *T. vaginalis* Parallels the Typical IRP/IRP Mechanism

**Figure 11:**
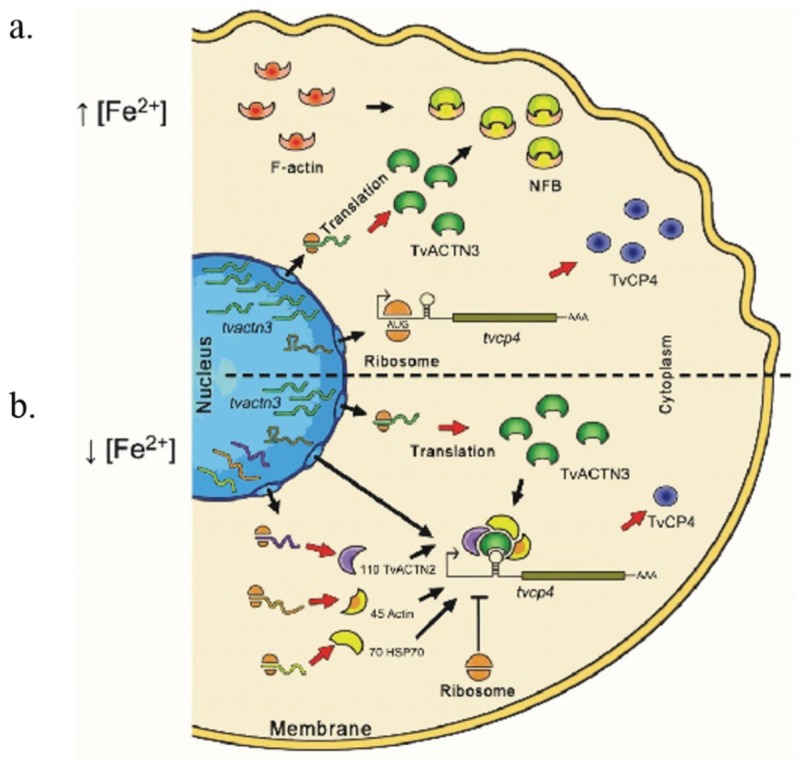
Hypothetical model for posttranscriptional iron regulation mediated by RNA-protein interactions in *T. vaginalis* using the *tvcp4* gene as a model. (**a**) Under iron-rich condition no RPC formation is observed due to that TvACTN3 and TvACT interact showing their typical functions as cytoskeleton proteins and TvHSP70 is degraded possible through the ubiquitin-proteasome pathway; thus, the *tvcp4* mRNA is translated; (**b**) Under iron-depleted conditions, TvACTN3 and TvACT do not interact, TvHSP70 is stable and all proteins moonlight by acquiring a new function as RNA-binding proteins that interact with IRE-like *tvcp4* hairpin structure and blocking translation of *tvcp4* mRNA.

According to the results presented in this review, we propose a hypothetical model of a parallel iron posttranscriptional regulatory mechanism mediated by RNA-protein interactions under iron-restricted conditions in *T. vaginalis* using the *tvcp4* gene as a model (Figure 11).

### 5.1. Possible Events Occurring under High Iron Conditions with tvcp4 Expression

The *tvhsp70-4*, *tvactn2*, *tvactn3*, *and tvact* mRNAs are expressed under high iron conditions (Figure 11a). However, less TvHSP70-4 is detected. Differences in the amount of TvHSP70-4 protein could be due to an increase in the level of ubiquitination under high iron conditions that could promote its degradation probably through the proteasome pathway, as occurs with IRP-2 [31,32]. *T. vaginalis* has all the elements of the proteasome mediated degradative machinery [85,89]. Moreover, under high iron conditions *tvactn2* and *tvactn3* and some of the *tvact* genes are overexpressed and TvACTN3 functions as an actin-binding protein that promotes the polymerization and stabilization of actin filaments [85,112,120] (Table 3). Therefore, none of the four cytoplasmic proteins (TvHSP70-4, TvACTN2, TvACTN3, and TvACT) described in here will function as an RNA-binding protein under high iron conditions.

For trichomonad IRE-like mRNA structures such as IRE-like *tvcp4* located in the 5′ region of the *tvcp4* mRNA, the gene encoding the TvCP4 protein was shown to be transcribed and its mRNA was shown to be translated under high iron conditions. Thus, we postulate that the *tvcp4* mRNA translation may be caused by the lack of RPC formation in its 5′ region hairpin IRE-like structure possibly due to the degradation of TvHSP70 and the switching to the typical function of the other two cytoskeleton proteins involved: Actinin and actin associated to form actin-actinin clusters under high iron conditions (Figure 9), likely aiding in actin polymerization.

### 5.2. Possible Events Occurring under Low Iron Conditions with tvcp4 Expression

Under low iron conditions (Figure 11b), the four proteins (TvHSP70-4, TvACTN2, TvACTN3 and TvACT) may function as RNA-binding proteins: TvACTN3 is not linked to actin in the actin clusters and is diffused in the cytoplasm and on the periphery of the nucleus (by an unknown mechanism), whereas TvHSP70 is also found near the protein synthesis machinery in the endoplasmic reticulum. Thus, the recently formed RPC may interact with the IRE-like structure located in the 5' region of the *tvcp4* mRNA, blocking its translation. The same phenomenon could be occurring with any other mRNA regulated by iron that possesses a hairpin structure similar to IRE-like *tvcp4* or IRE-like *tvcp12* and may help iron to regulate its expression at the posttranscriptional level. Work is in progress to search for mRNAs with IRE-like hairpin structures that are differentially regulated by iron at the posttranscriptional level.

### 5.3. Possible Events Occurring under Low and High Iron Conditions with Tvcp12 Expression

Considering that IRE-like *tvcp12* could bind the same multifunctional proteins as IRE-like *tvcp4*, we propose that a similar hypothetical mechanism described in Figure 11 could also apply. The same RNA-binding proteins will interact with IRE-like *tvcp12* hairpin structure located at the 3'-UTR of the *tvcp12* mRNA in the absence of iron, and the outcomes will be *tvcp12* mRNA stabilization and TvCP12 translation under low iron conditions. In contrast, because no RNA-protein complex will be formed under high iron conditions, *tvcp12* mRNA will be degraded and no TvCP12 protein will be observed.

### 5.4. A Comparative Analysis of Trichomonad RNA-Binding Proteins with Typical IRPs

A comparison between the RNA-binding proteins found in *T. vaginalis* and other parasites with the canonical IRPs is shown in Table 4. All proteins are localized in the cytoplasm and interact with RNA in the absence of iron. The multifunctional proteins described in Table 4 behave as RNA-binding proteins under low iron conditions and show their typical functions under high iron conditions.

**Table 4:** Comparative analysis of iron regulatory protein (IRP)-1, IRP-2 and protozoan IRP-like proteins with *T. vaginalis* atypical RNA-binding proteins described in here.

Characteristic	IRP-1	IRP-2	pfIRPa ^a^	TvHSP70 ^b^	TvACTN3 ^c^	TvACTN2 ^d^	TvACT ^d^
RNA-interaction under iron-depleted condition	Yes	Yes	Yes	Yes	Yes	ND	ND
Reducing agent dependence	Yes	No	Yes	No	No	No	No
Cytoplasmic localization	Yes	Yes	Yes	Yes	Yes	Yes	Yes
Functions	RBP Aconitase	RBP NP	RBP Aconitase	RBP Chaperone	RBP Actin-binding protein	NC Actin-binding protein	NC Cytoskeleton filaments
Molecular Weight (kDa)	98	105	103	70	135	110	45

ND: not determined; NP: not present or other function; NC: not confirmed. ^a^ [75]; ^b^ Arroyo *et al.*, 2015 under revision; ^c^ [120]; ^d^ This review.

Interestingly, the experimentally identified RNA-binding proteins in *T. vaginalis* belong to multigene families where only a few members are expressed under different environmental conditions, a very common behavior in trichomonads*,* as in the case of the CP families. Several theories are being explored to explain and understand it [17]; one possible explanation could be that only few environmental conditions have been studied thus far.

## 6. Conclusions and Future Directions

Most pathogens require iron to survive and express their virulence factors. Microorganisms have developed a number of mechanisms for iron acquisition from their host cells, competing for iron with other organisms sharing the same host niche, and developing specialized systems for iron uptake such as the production of siderophores to bind directly Fe(III) or specific ligands to entrap siderophilins and the usage of proteases and reductases to cleave and reduce bound iron to Fe(II) for internalization by iron transporters [161,162]. However, an excess of iron is also toxic. Thus, mammals regulate cellular iron homeostasis through an IRP/IRE mechanism. IRPs belonging to the aconitase family interact with IREs located at the 5'- or 3'-UTR of several mRNAs under low iron conditions. As we mentioned above, *T. vaginalis* lacks the Krebs cycle and aconitase activity. Thus, in this review we propose that *T. vaginalis*, an early evolving protist parasite, may use other alternative and multifunctional cytoplasmic proteins to posttranscriptionally regulate the expression of virulence factors by iron through interactions with IRE-like hairpin structures located in the 5'- or 3'-UTR in the absence of iron. The multifunctional proteins TvHSP70, TvACTN2, TvACTN3, and TvACT, behave as RNA-binding proteins that specifically interact with the *T. vaginalis* IRE-like hairpins and may help to mediate iron-regulated gene expression. Taken together, data suggest the existence of a parallel posttranscriptional mechanism mediated by RNA-protein interactions, such as in the IRE/IRP system, suggesting that trichomonad IRE-like sequences are able to interact with the multifunctional proteins (TvHSP70, TvACTN2, TvACTN3, and TvACT) and with the typical hIRP. Thus, trichomonad IRE-like *tvcp4* and IRE-like *tvcp12* hairpin structures may be the ancestral forms of the RNA stem-loop structures of the IRE/IRP system. Interestingly, future work will aim to identify other mRNAs differentially regulated by iron that also share similar RNA hairpin structures described herein and to demonstrate that the atypical posttranscriptional iron-regulated mechanism occurs *in vivo* in *T. vaginalis* grown under different iron conditions as determined by *in vitro* functional assays.
